# Tomographic evaluation of the influence of the placement of a collagen membrane subjacent to the sinus mucosa during maxillary sinus floor augmentation: a randomized clinical trial

**DOI:** 10.1186/s40729-019-0183-5

**Published:** 2019-08-19

**Authors:** Atsuya Hirota, Niklaus P. Lang, Mauro Ferri, Natalia Fortich Mesa, Karol Ali Apaza Alccayhuaman, Daniele Botticelli

**Affiliations:** 1 Department of Oral Implantology, Osaka Dental University, Osaka, Japan; 2 ARDEC Academy, Ariminum Odontologica, 47923 Rimini, Italy; 3 School of Dental Medicine, University of Berne, Berne, Switzerland; 4 Corporación Universitaria Rafael Núñez, Cartagena de Indias, Colombia

**Keywords:** Maxillary sinus floor augmentation, Collagen membrane, Xenograft, Oral surgical procedures

## Abstract

**Aim:**

To study the influence of a collagen membrane placed subjacent to the sinus mucosa on the dimensional changes of augmented maxillary sinus floor.

**Methods:**

Twenty patients were recruited in the study and randomly assigned to two groups. After the elevation of the maxillary sinus mucosa, a collagen membrane with standardized dimensions was placed at the test sites subjacent to the sinus mucosa and the elevated space was filled with a xenograft, both at test and control sites. A collagen membrane was then used to cover the antrostomy at both sites, and sutures were applied to close the wounds. Cone beam computed tomographies (CBCTs) were taken for all patients before surgery (T0), after 1 week from sinus floor augmentation (T1), and after 9 months of healing (T2). Dimensional changes over time of soft and hard tissues were evaluated on the CBCTs.

**Results:**

After 1 week of healing, the sinus floor was elevated by 10.0 ± 2.8 mm and 10.6 ± 2.5 mm at the no-membrane and membrane groups, respectively. After 9 months of healing, a similar reduction of the height was observed in both groups, providing a total vertical augmentation of 8.6 ± 2.8 mm at the no-membrane sites and 9.1 ± 3.1 mm at the membrane sites. After 9 months of healing, the hard tissues subjacent to the sinus mucosa appeared to be partially corticalized in three patients in the no-membrane group and in six patients in the membrane group.

**Conclusions:**

The use of collagen membranes subjacent to the sinus mucosa did not influence the dimensional variations of the augmented regions and the clinical outcomes after 9 months of healing also in absence of perforations.

## Introduction

The perforation of the sinus mucosa during sinus floor augmentation is a complication that has been reported in several clinical [1–4] and experimental studies [5, 6], with an occurrence that varies between 10 and 55% [1, 2]. Dislodgement of the biomaterial and sinusitis may be the logical consequence of the perforation [2]. Small perforations may not require treatments because the margin of the perforation may collapse and close the defect [7]. Perforations of larger dimensions may be closed using sutures [1, 8, 9] or fibrin glue [1, 8, 10]. Collagen membranes were also recommended to protect perforations of the sinus mucosa [3, 4, 11–15].

Several experimental studies have been performed to evaluate histologically the influence of a collagen membrane placed subjacent to the sinus mucosa in the absence [5, 15] or presence of perforations [16], and no differences were seen in the healing outcomes. However, there is still a lack of clinical information about how the placement of a collagen membrane between the sinus mucosa and the graft may influence dimensional variations and healing at augmented maxillary sinuses. Hence, the aim of this randomized clinical trial was to study the influence of a collagen membrane placed subjacent to the sinus mucosa on the dimensional changes of augmented maxillary sinus floors.

The null hypothesis was that of no difference between the dimensional variations over time of the augmented regions applying or not a collagen membrane subjacent to the sinus mucosa.

## Material and methods

The protocol was approved by the Ethical Committee of the University Corporation Rafael Núñez, Cartagena de Indias, Colombia (protocol #03-2015; 4 December 2015), and the Declaration of Helsinki on medical protocols and ethics was applied. The patients were informed about the procedures and the possible complications and signed the informed consent. The CONSORT checklist was followed for this study (http://www.consort-statement.org/). The present RCT was recorded at the ClinicalTrials.gov (https://clinicaltrials.gov/) and received the identifier number NCT03902457.

### Study population

In this randomized clinical trial, patient recruitment, surgeries, and follow-ups were performed at the University Corporation Rafael Núñez, in Cartagena de Indias (Colombia). To calculate the sample size, the data from a study that assessed the variations over time in height of the augmented sinus floors were used [17]. It was calculated that an *n* = 10 was sufficient to show statistically significant differences in change of height over time, if any difference existed. An author not involved in the surgeries performed the randomization (MF). The assignments were sealed in opaque envelopes that were opened after the completion of the elevation of the sinus mucosa.

The inclusion criteria for the participants were as follows:

1. Presence of an edentulous zone in the posterior maxilla
2. Height of the sinus floor of about 4 mm or less
3. Need for a prosthetic restoration supported by implants in the distal segment of the maxilla
4. ≥ 21 years old
5. Being in good general health with no contraindications for oral surgical procedures
6. Not being pregnant

The exclusion criteria were as follows:

1. Presence of a systemic disorder
2. Chemotherapic or radiotherapeutic treatments in the past or in progress
3. Smokers with declared smoking of > 10 cigarettes per day
4. Acute or a chronic sinusitis
5. Bone augmentation procedures in the zone of interest

### Biomaterial used

The xenograft used as filler material was a collagenated corticocancellous porcine bone granules (Gen-Os, 250–1000 μm, OsteoBiol, Tecnoss, Giaveno, Italy).

The membrane used subjacent the sinus mucosa at the test sites as well as to cover the antrostomy at both test and control sites was an equine collagen membrane (Evolution, 0.3 mm, OsteoBiol, Tecnoss, Giaveno, Italy).

### Clinical procedures

The lateral wall of the maxillary sinus was exposed, and an antrostomy of about 6 mm in height and 10 mm long was prepared grinding the bone with a round diamond insert (SFS 109 029, Komet-Brasseler-GmbH, Germany), mounted on a sonic-air surgical instrument (Sonosurgery® TKD, Calenzano, Fi, Italy). The sinus mucosa was elevated, and at the test sites, a collagen membrane of standardized dimensions (9 × 13 mm) was placed subjacent the sinus mucosa. Subsequently, the elevated space was filled both at the test and control sites with the xenograft soaked with saline. A collagen membrane was used to protect the antrostomies, and the soft tissue wounds were sutured.

Amoxicillin 875 mg with clavulanic acid 125 mg twice a day for 6 days, analgesic drugs as needed, and mouth rinses with 0.12% chlorhexidine three times a day for 10 days were recommended. The sutures were removed after 7 days, and the patients were enrolled in a maintenance program for the full extent of the study.

The definitive implants (Sweden & Martina, Due Carrare, Padua, Italy) were installed 9 months after sinus floor elevation.

### CBCT imaging procedures

Cone beam computed tomographies (CBCTs) were taken at three different periods: before the sinus floor elevation (T0), and 1 week (T1) and 9 months (T2) after surgery.

### CBCT imaging analyses

**Fig. 1:**
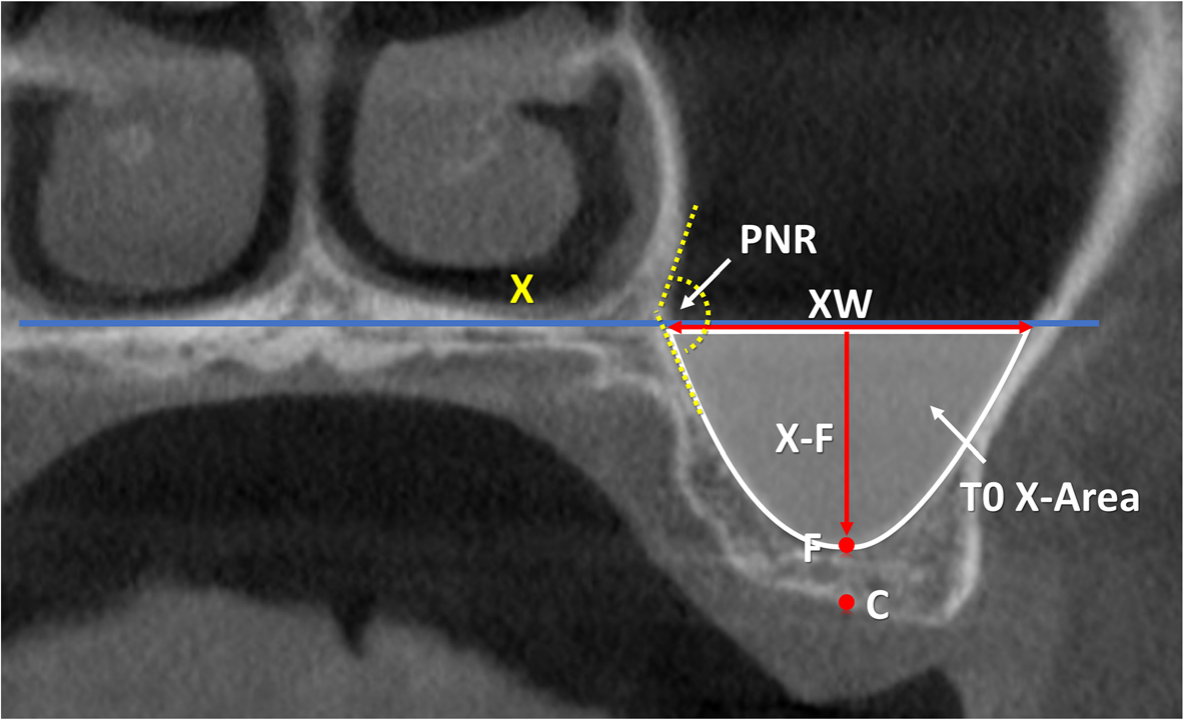
T0, coronal view of a no-membrane CBCT; X, line drawn following the floor of the nose; C, center of the bony crest; F, base of the sinus floor; PNR, palatal-nasal recess. X-F, nasal floor height; XW (sinus width), distance evaluated on the line X between the two intersection points with the medial and lateral sinus bone walls; T0 X-Area, area delimited by the sinus bone walls and the line X. The PNR angle is indicated in yellow

**Fig. 2:**
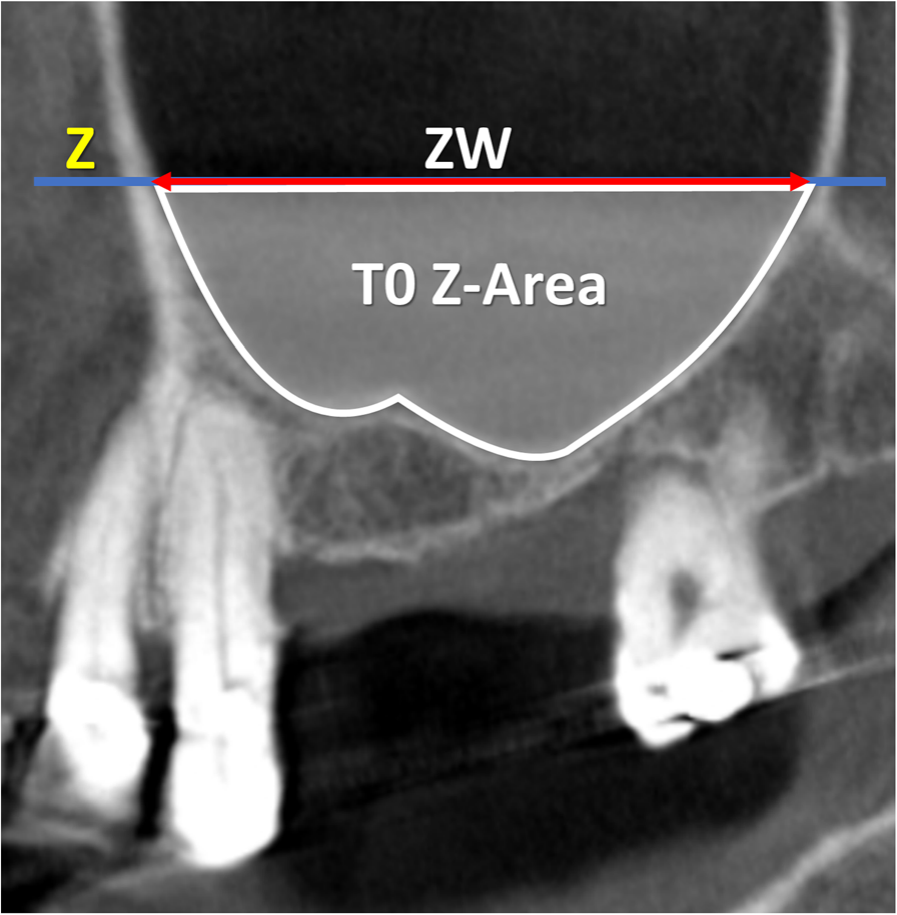
T0, lateral view of a no-membrane CBCT; Z, line drawn following the floor of the nose; ZW (sinus extension), distance evaluated on the line Z between the two intersection points with the mesial and distal sinus bone walls; T0 Z-Area, area delimited by the sinus bone walls and the line Z

**Fig. 3:**
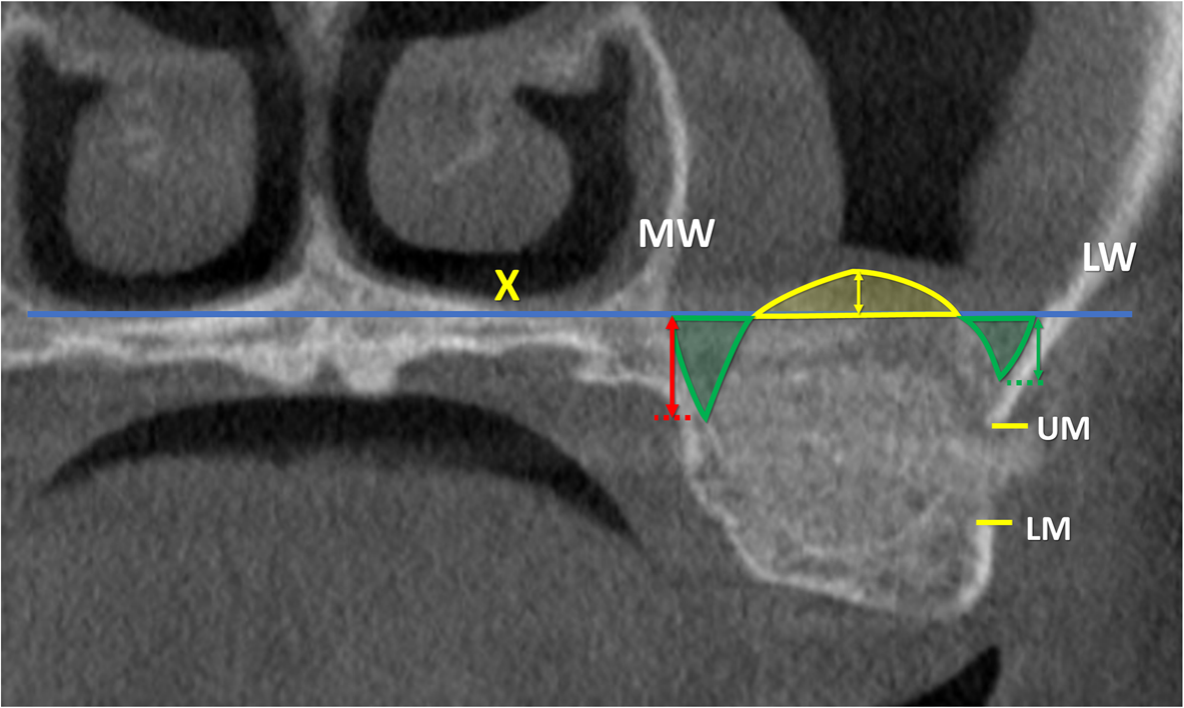
T1, coronal view of a no-membrane CBCT; X, line drawn following the floor of the nose; F, base of the sinus floor; MW, medial wall of the sinus; LW, lateral wall of the sinus; UM, upper margin of the antrostomy; LM, lower margin of the antrostomy. Exceeding area (bordered in yellow), area above the line X filled with biomaterial/ bone tissue. Residual areas (bordered in green), the areas below the line X not filled with biomaterial/ bone tissue. Floor augmentation heights at the lateral (green arrow), middle (yellow arrow), and medial (red arrow) aspects

**Fig. 4:**
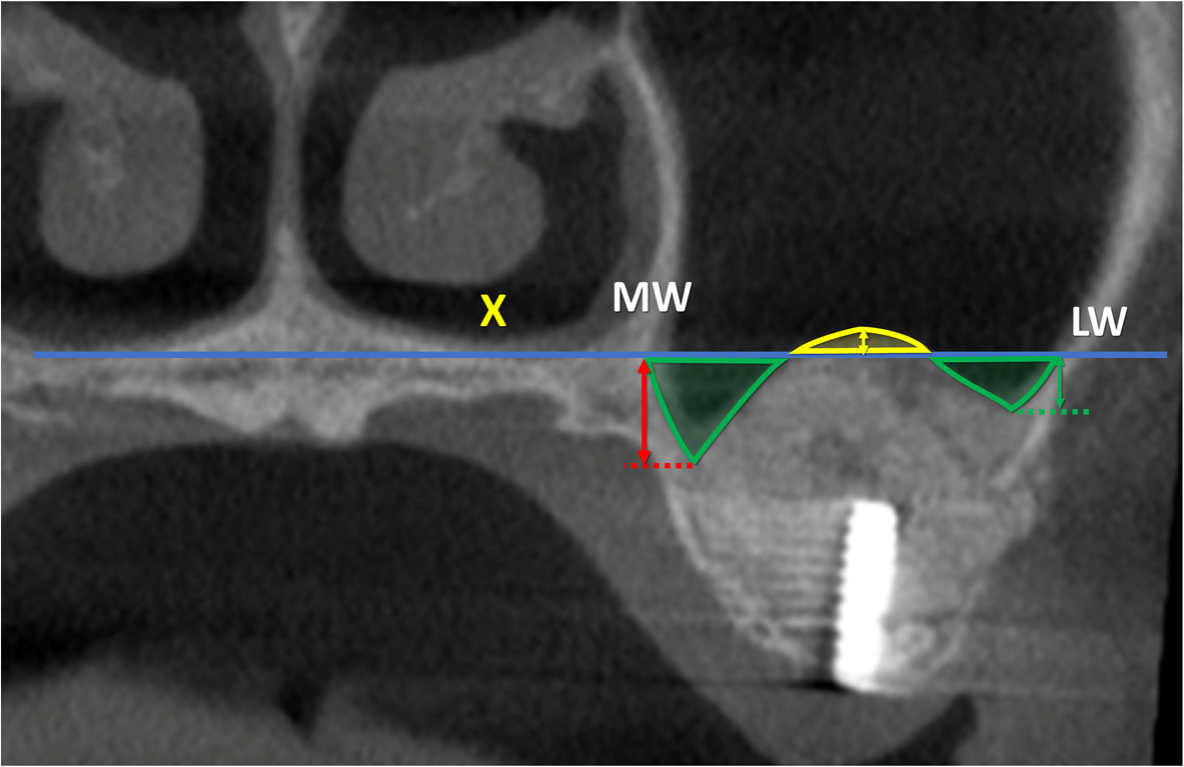
T2, coronal view of a no-membrane CBCT; X, line drawn following the floor of the nose; MW, medial wall of the sinus; LW, lateral wall of the sinus; Exceeding area (bordered in yellow), area above the line X filled with biomaterial/ bone tissue. Residual areas (bordered in green), the area below the line X not filled with biomaterial/ bone tissue. Floor augmentation heights at the lateral (green arrow), middle (yellow arrow), and medial (red arrow) aspects

All radiographic evaluations were performed with the software i-Dixel 2.0 (J. Morita Corporation, Kyoto, Japan) following the protocol illustrated in previously published papers (Table 1) [3, 4]. As references, a line was drawn following the floor of the nose both in the coronal (*X*-axis; Fig. 1) and in the lateral views (*Z*-axis; Fig. 2). A series of parameters were evaluated at the various periods. The height of the augmentation at the medial, middle, and lateral aspects (Figs. 3 and 4) was obtained subtracting (if located below the *X*-axis) or adding (if located above the *X*-axis) to the sinus height (distance *X*–*F*) the distance between the most coronal position of the hard tissue at the respective aspects. The augmented area was calculated adding the exceeding area or subtracting the residual areas to the *X*-area (Figs. 3 and 4). The variations in height and area of the elevated region were evaluated comparing the CBCTs taken at the three periods, T0, T1, and T2.

**Table 1:** Measurements performed on the CBCTs at the various periods of evaluation

T0	C-F	Bone crest height
T0	X-F	Sinus height
T0	PSAA-C	Distance between the posterior superior alveolar artery and C
T0	PSAA diameter	Posterior superior alveolar artery diameter
T0	PNR angle	Palatal-nasal recess angle (angle formed by the palatal and nasal bone wall)
T0	X-W	Sinus width (distance between the lateral and medial wall on the *X*-axis)
T0	ZW	Sinus length (distance from the mesial and distal bone walls on the *Z*-axis)
T0	X-area	Area delimited by *X*-axis and sinus bone walls
T0	Z-area	Area delimited by *Z*-axis and sinus bone walls
T0, T1, T2	MT	Mucosa thickness
T1	LM-F	Balcony height
T1	UM-LM	Antrostomy height
T1, T2	X-MW	Distance between *X*-axis and the most coronal position of the hard tissue at the medial sinus bone wall
T1, T2	X-MA	Distance between *X*-axis and the most coronal position of the hard tissue at the middle aspect
T1, T2	X-LW	Distance between *X*-axis and the most coronal position of the hard tissue at the lateral sinus bone wall
T1, T2	X-EA	Elevated are at T1 and T2 in the coronal view
T1, T2	Z-EA	Elevated are at T1 and T2 in the lateral view
T1, T2	E-area	Exceeding area
T1, T2	R-area	Residual area

### Data analysis

The primary outcome variable was the change in height of the elevated sinus floor zone between 1 week and 9 months. The secondary outcome variable was the area variation of the elevated zone between 1 week and 9 months. The radiographic measurements were performed twice by a well-trained researcher that was blinded about the differences in the protocols (KAAA). Mean values were obtained between the two measurements and used for analyses.

Mean values and standard deviations (SD) were calculated for each outcome variable. Differences between the membrane and no-membrane sites were analyzed with the Mann-Whitney test using the IBM SPSS Statistics software (IBM, Chicago, IL, USA). The level of significance was set at α = 0.05.

## Results

**Fig. 5:**
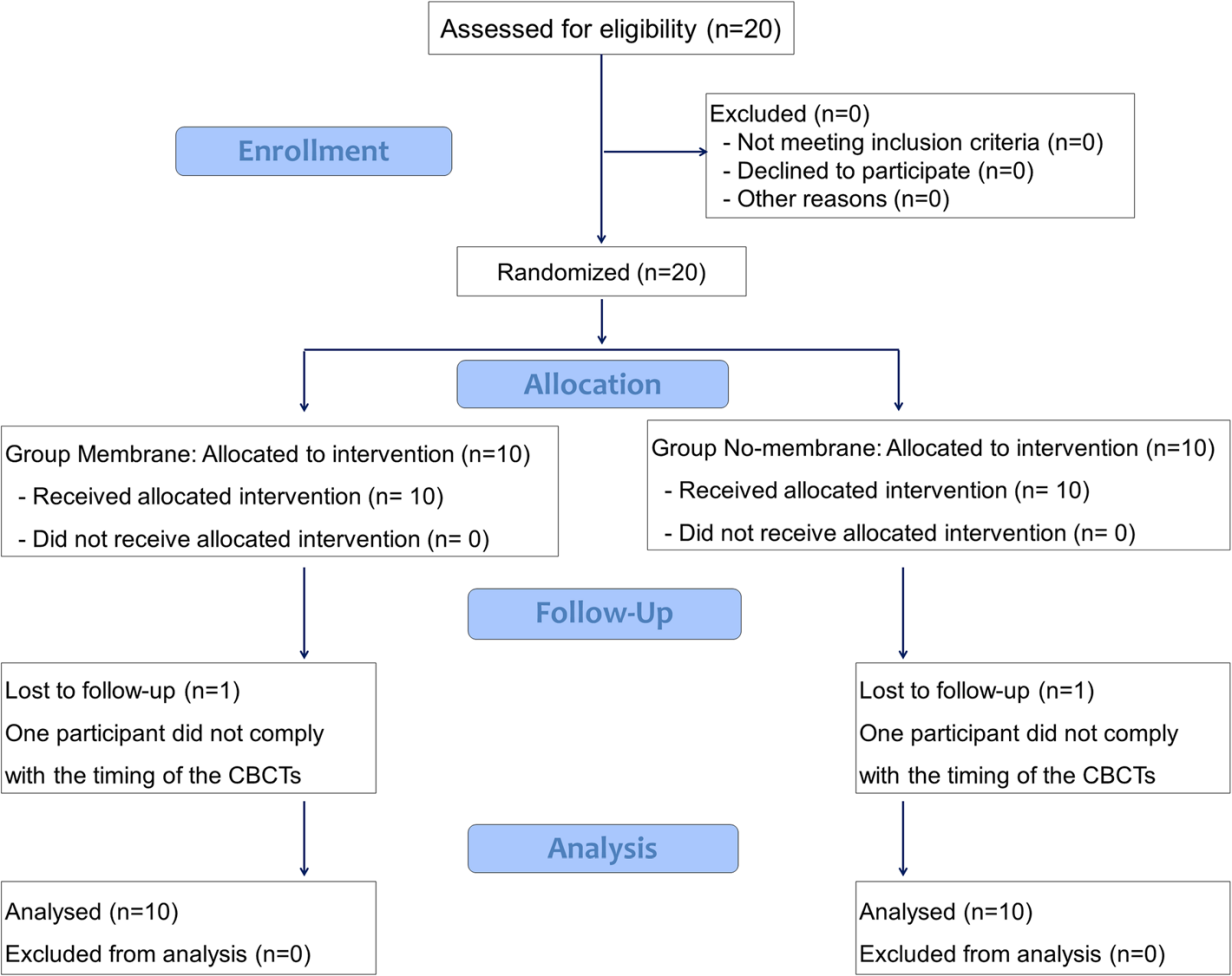
CONSORT 2010 flow diagram

The study initiated in February 2016 and ended in December 2018. Twenty participants were included. One perforation of ~ 5 mm of diameter occurred at a membrane site. The perforation was covered with the collagen membrane, and the patients were maintained in the study. No patients presented complications during the period of healing. However, one patient in each group did not comply with the timing of the x-rays so that the CBCTs at T9 (9 months) were not available and an *n* = 9 was reached for both groups (Fig. 5). Five females and four males, 55.8 ± 9.3 years old, formed the no-membrane group while six females and three males, 53.4 ± 9.7 years old, were included in the membrane group (Table 2).

**Table 2:** Demographic and clinical data (*n* = 9)

	Sex	Age (years)	Smokers	Side
No-membrane	5 females, 4 males	55.8 ± 9.3	None	5 right, 4 left
Membrane	6 females, 3 males	53.4 ± 9.7	None	6 right, 3 left

*p* < 0.05

### CBCT imaging evaluation

The anatomical data evaluated at T0 were reported in Table 3 and were related to the bone crest height, the distance between the *X*-axis and the base of the sinus, the width of the sinus evaluated on the *X*-axis, the position and diameter of the intraosseous anastomosis (that connect to the posterior superior alveolar artery to the infraorbital artery), and the angle of the palatal-nasal recess. Moreover, the height of balcony and antrostomy was also reported.

**Table 3:** Radiographic anatomical data in the coronal view taken at different periods

	Bone crest height (C-F) at T0	Sinus height (X-F) at T0	Sinus width (XW) at T0	PSAA height (PSAA-C) at T0	PSAA diameter at T0	PNR angle	Balcony height (LM-F) at T1	Window height (LM-UM) at T1
No-membrane	3.1 ± 0.7	10.7 ± 2.4	16.0 ± 4.2	16.0 ± 3.4	1.4 ± 0.4	123.0 ± 27.2	3.8 ± 0.8	5.7 ± 1.1
Membrane	3.4 ± 1.1	9.8 ± 2.6	17.1 ± 2.3	16.9 ± 2.4	1.1 ± 0.3	129.2 ± 12.6	4.3 ± 1.0	5.9 ± 0.3

*T0* before surgery, *T1* 1 week, *T2* 9 months, *PSAA* posterior superior alveolar artery. Data in millimeters. *p* < 0.05

**Fig. 6:**
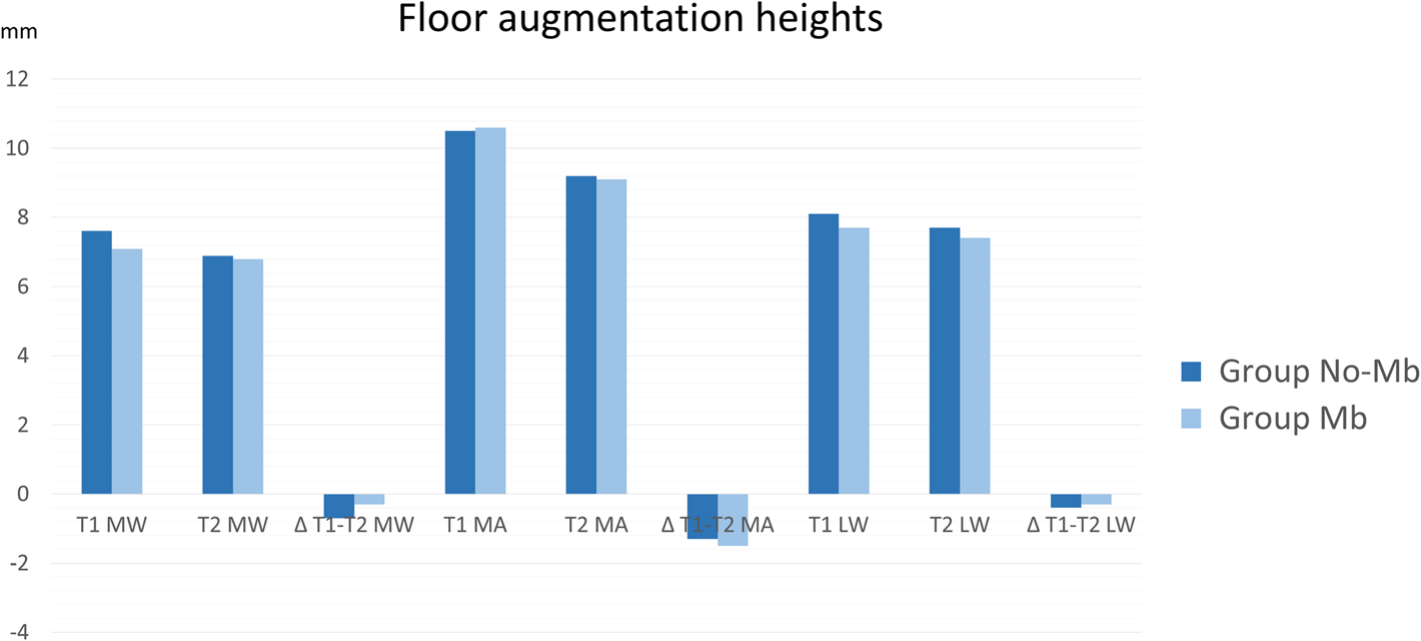
Graph representing floor augmentation and loss (Δ) of heights between 1 week (T1) and 9 months (T2) in the coronal plane evaluated at the medial (MW), middle (MA), and lateral (LW) walls of the sinus. Data in millimeters

In the middle aspect, the elevation of the sinus mucosa was similar in both groups, being 10.0 ± 2.8 mm at the no-membrane and 10.6 ± 2.5 mm at the membrane sites (Table 4; Fig. 6). A reduction in height of 1.4 ± 1.2 mm and 1.4 ± 2.3 mm was observed in the no-membrane and membrane sites, respectively. The medial and lateral aspects were elevated to a lesser extent in respect to the middle aspect, providing a dome shape of the elevated region.

**Table 4:** Floor augmentation heights in the coronal view evaluated at the medial, middle, and lateral aspects of the sinus at the various periods of observation

	Medial wall			Middle aspect			Lateral wall		
	T1	T2	Δ T1–T2	T1	T2	Δ T1–T2	T1	T2	Δ T1–T2
No-Membrane	7.4 ± 2.6	6.5 ± 1.6	− 0.9 ±1 6	10.0 ± 2.8	8.6 ± 2.8	− 1.4 ± 1.2	7.5 ± 2.7	7.0 ± 2.7	− 0.5 ± 0.8
Membrane	7.1 ± 2.6	6.8 ± 2.1	− 0.3 ± 1.4	10.6 ± 2.5	9.1 ± 3.1	− 1.4 ± 2.3	7.7 ± 1.7	7.4 ± 1.9	− 0.3 ± 1.1

*T1* 1 week, *T2* 9 months, *Δ* difference. Data in millimeters. *p* < 0.05

In the no-membrane sites, between 1 week and 9 months of healing, the areas were reduced of ~ 20%, both in the coronal and lateral view (Table 5). In the membrane sites, 14% of reduction of the areas was observed both at the coronal and lateral views.

**Table 5:** Areas (in mm^2^) in the *X* and *Z* planes at the various periods evaluated (T1 = 1 week; T2 = 9 months) and shrinkage in mm^2^ and percentages (%) of the elevated space between 1 week and 9 months in the coronal and lateral planes

		*X*-area and T0 *Z*-area at T0	Elevated areas at T1	Elevated areas at T2	Δ T1–T2 (mm^2^)	Δ T1–T2 (%)
Coronal view	No-membrane	123.0 ± 27.2	84.5 ± 38.2	68.7 ± 36.4	− 15.7 ± 14.1	− 20.2 ± 16.8
	Membrane	129.2 ± 12.6	92.8 ± 14.8	78.1 ± 19.5	− 14.6 ± 23.3	− 14.4 ± 21.9
Lateral view	No-membrane	173.3 ± 92.3	129.6 ± 57.6	105.6 ± 56.8	− 24.0 ± 21.7	− 19.9 ± 22.0
	Membrane	175.7 ± 33.2	124.4 ± 30.7	103.7 ± 25.7	− 20.6 ± 22.5	− 14.1 ± 17.4

*p* < 0.05. *Δ* difference

At T0, the sinus mucosa was thicker at the no-membrane compared to the membrane sites, even though no statistically significant differences were disclosed (Table 6).

**Table 6:** Sinus mucosa thickness at the various periods of evaluation

	T0	T1	T2	Δ T1–T0	Δ T2–T1	Δ T2–T0
No-membrane	5.6 ± 8.4	10.5 ± 7.0	1.3 ± 0.9	4.8 ± 7.6	− 9.1 ± 6.4	− 4.3 ± 7.7
Membrane	2.1 ± 2.1	6.3 ± 6.9	1.5 ± 1.2	4.1 ± 7.8	− 4.8 ± 6.9	− 0.6 ± 2.4

None of the differences was statistically significant between group A and group B. *Δ* difference, *T1* 1 week, *T2* 9 months. Data in millimeters

*p* < 0.05

After 1 week, the mucosa increased in thickness by ~ 4–5 mm in both sites, while after 9 months, the thickness was reduced to 1.3–1.5 mm in both groups.

The hard tissues underneath the sinus mucosa appeared to be partially corticalized in three cases in the no-membrane group and in six cases in the membrane group. The antrostomy appeared to be closed in four cases in the no-membrane group and in six cases in the membrane group.

## Discussion

The aim of the present study was to evaluate the influence of a collagen membrane placed subjacent the sinus mucosa on the dimensional changes of augmented maxillary sinus floors. No statistically significant differences were found between the sites with and without the placement of a collagen membrane.

The influence on healing of the placement of a collagen membrane subjacent the sinus mucosa was studied in sheep and rabbits, both in perforated [15] or not perforated sinus mucosae [5, 15]. Also in those studies, no differences were found between the sites with or without the collagen membrane.

The results from the present study are not in agreement with those from another experimental study in rabbits [18]. In that experiment, a perforation was intentionally produced in the sinus mucosa of the test sites and a collagen membrane was placed as protection subjacent to the sinus mucosa. The healing was evaluated after 4 and 8 weeks from surgery. It was concluded that the placement of a collagen membrane delayed bone formation within the sinus. However, it must be considered that, in that study, the collagen membrane was extended to cover also the sinus bone walls. This may have prevented bone formation from the sinus bone walls within the elevated space.

Other variables have been studied to assess their influence on the dimensional changes over time of the elevated sinus floor. In an RCT on sinus floor elevation using a lateral access,^4^ the antrostomy was prepared either close to the level of the floor in the test group, or about 3.5 mm above in the control group. Similarly to the present study, also in that study, a collagenated corticocancellous porcine bone was used to fill the elevated space. One week after floor elevation, the gain in height in the middle region of the elevated space was 9.9 mm in the test sites, and 10.9 mm in the control sites. After 9 months of healing, a total gain of 7.7 mm at the test, and 9.4 mm at the control sites was obtained, indicating a higher total gain at the control sites. It was concluded that the position of the antrostomy in relation to the sinus floor might affect the height of the augmentation. In the present study, the base of the antrostomy was placed at about 4 mm from the sinus floor. The augmentation evaluated after 1 week was about 10–10.6 mm in both groups. After 9 months following surgery, a total gain of about 9 mm was obtained, similar to that observed in the control group of the study discussed previously [4]. The loss of height might be attributed to the resorption of the biomaterial [5, 6] or a displacement of the biomaterial, especially through the antrostomy [19].

In another RCT, the dimension of the antrostomy was tested [3]. A xenograft, similar to that used in the present study, was applied for sinus floor elevation. The small antrostomy measured ~ 50 mm^2^, while the large antrostomy was ~ 100 mm^2^. The final gain in height of the sinus floor was about 10 mm at the small antrostomy and about 9 mm at the large antrostomy. A higher loss of vertical dimension after 9 months of healing was registered at the large (− 3 mm) compared to the small antrostomy (− 2.1 mm). This is in agreement with the results from the present RCT in which the dimension of the antrostomy was ~ 60 mm^2^ in both groups and a minor loss of 1.4 mm was observed.

In the present study, the base of the nose was used as reference for the *X*-axis in the coronal view. This axis roughly corresponded to the palatal-nasal recess (PNR). Depending on the distance between the *X*-axis and the sinus floor, the elevation of the sinus mucosa beyond the PNR might be required. In such cases, the angle formed at the PNR by the palatal and nasal bone walls might represent a risk for mucosa perforation when it is < 90° [20]. In the present study, only in two cases, both in the membrane group, the sinus mucosa at the palatal aspect was elevated beyond the PNR. In that case, the PNR angle was ≥ 130° so that the risk of perforations was low. Moreover, the perforation that occurred at the test site was produced during the detachment of the mucosa around the antrostomy and not when the mucosa was separated from the palatal aspect.

Bone walls are the main source of new bone formation [5, 21–23] so that it is important to detach properly the mucosa also at the palatal aspect. For the same reason, it might be important to place the antrostomy cranially to the base of the sinus to leave a “balcony” of bone that might support bone formation within an important region for implant installation. It should be considered that larger antrostomies might eliminate large portions of the lateral bone wall, resulting in a reduced source for new bone formation.

The position and the dimension of the anastomosis that connects the posterior superior alveolar artery to the infraorbital artery have to be considered to decide dimensions and position of the antrostomy. In the present study, this anastomosis was located at ~ 16–17 mm cranially to bone crest. The mean height of the balcony was ~ 4 mm, the mean height of the antrostomy was ~ 6 mm, and that of the bony crest was ~ 3.1–3.4 mm. This, in turn, means that the upper border of the antrostomy was located as a mean value at about 13–14 mm from the bone crest that is 2–3 mm from the intraosseous anastomosis. In the present study, the upper border of the antrostomy never reached the anastomosis in any patients. When the anastomosis is reached, a hemorrhagic event might occur that requires an additional surgical treatment [13], especially if the diameter of the anastomosis is >1 mm. In the present study, the mean diameter of the anastomosis was 1.4 mm at the no-membrane sites and 1.1 mm at the membrane sites, and only four anastomoses presented a diameter < 1 mm. To reduce the incidence of damages to the anastomosis and perforations of the sinus mucosa, a round diamond insert mounted on a sonic-air surgical device was used to prepare the antrostomy. The use of an ultrasonic [24] or a sonic instrument [3, 4, 25, 26] has been shown to reduce the incidence of damages of soft tissues, as well as the perforations of the mucosa, especially if used to grind the lateral bony wall [27].

In the present study, 1 week after the surgery, the thickness of the mucosa increased by ~ 4–5 mm in both groups. This agrees with other studies that showed such an event, due to edema and bleeding [3, 4, 19, 28]. The thickness of the sinus mucosa after 9 months of healing was similar in both groups (1.3–1.5 mm). These outcomes are in agreement with other studies that reported a return to a normal thickness of the mucosa after healing [3, 4, 19, 28].

As limitations of the present study, it may be mentioned that there is a lack of long-term clinical outcome.

In conclusion, the use of a collagen membrane subjacent to the sinus mucosa did not influence the dimensional variations of the augmented regions and the clinical outcome after 9 months of healing also in the absence of perforations.

## Data Availability

The datasets used or analyzed during the current study are available from the corresponding author on reasonable request.

## Abbreviations

CBCT: Cone beam computed tomography
RCT: Randomized clinical trial
SD: Standard deviation
T0: Baseline, time of sinus floor elevation
T1: One week after baseline
T2: Nine months after surgery
Δ: Difference
